# Connectivity, not region-intrinsic properties, predicts regional vulnerability to progressive tau pathology in mouse models of disease

**DOI:** 10.1186/s40478-017-0459-z

**Published:** 2017-08-14

**Authors:** Chris Mezias, Eve LoCastro, Chuying Xia, Ashish Raj

**Affiliations:** 1 Department of Neuroscience, Weill Cornell Medicine of Cornell University, New York, USA; 2 Department of Radiology, Weill Cornell Medicine of Cornell University, New York, USA

## Abstract

Spatiotemporal tau pathology progression is regarded as highly stereotyped within each type of degenerative condition. For instance, AD has a progression of tau pathology consistently beginning in the entorhinal cortex, the locus coeruleus, and other nearby noradrenergic brainstem nuclei, before spreading to the rest of the limbic system as well as the cingulate and retrosplenial cortices. Proposed explanations for the consistent spatial patterns of tau pathology progression, as well as for why certain regions are selectively vulnerable to exhibiting pathology over the course of disease generally focus on transsynaptic spread proceeding via the brain’s anatomic connectivity network in a cell-independent manner or on cell-intrinsic properties that might render some cell populations or regions uniquely vulnerable. We test connectivity based explanations of spatiotemporal tau pathology progression and regional vulnerability against cell-intrinsic explanation, using regional gene expression profiles as a proxy. We find that across both exogenously seeded and non-seeded tauopathic mouse models, the connectivity network provides a better explanation than regional gene expression profiles, even when such profiles are limited to specific sets of tau risk-related genes only. Our results suggest that, regardless of the location of pathology initiation, tau pathology progression is well characterized by a model positing entirely cell-type and molecular environment independent transsynaptic spread via the mouse brain’s connectivity network. These results further suggest that regional vulnerability to tau pathology is mainly governed by connectivity with regions already exhibiting pathology, rather than by cell-intrinsic factors.

**Electronic supplementary material:**

The online version of this article (doi:10.1186/s40478-017-0459-z) contains supplementary material, which is available to authorized users.

## Introduction

Tauopathic degenerative conditions, such as Alzheimer’s Disease (AD), are united by exhibiting transregionally spreading proteinopathy, resulting in stereotyped spatiotemporal progression patterns [5, 30, 31]. The central questions are how transregional spread occurs, why the macroscale patterning of tau protein pathology progression seen in both patient [5] and mouse model [17] studies is so consistent, and what underlies regional vulnerability to tau pathology. Prior clinical and mouse model research suggests both anatomic connectivity [23] and molecular signatures of vulnerable regions, as reflected in regional gene expression profiles [12], might underlie transregional tau pathology progression. Connectivity based progression theories posit that spatiotemporal tau pathology development will follow fiber tracts, while cell intrinsic hypotheses assert that individual cellular factors, for which we use regional gene expression as a proxy, underwrite spatial pathology progression. Although the concept that propagation of tau pathology is determined by brain connectivity has been extensively studied and proved experimentally by different research groups [2, 9, 20], no previous study has directly and quantitatively tested the role of connectivity in comparison with cell intrinsic properties like regional gene expression.

Several lines of evidence using clinical and mouse model work support theories that give primacy to connectivity. Graph theoretic modeling on large sets of public patient data, in particular the network diffusion (ND) model [33] and the epidemic spread model [21], suggest that connectivity with regions already exhibiting pathology predicts the unfolding pattern of atrophy and amyloid deposition, respectively. These models, which used mathematical equations to predict the spread of pathology on the brain network, significantly recreate both the cross-sectional patterns of regional volumetric loss [33] and longitudinal volumetric loss and glucose metabolic deficits [34]. Observational data on the cellular localization of misfolded tau in transgenic mice indicates particularly heavy buildup at both post and presynaptic synapses [16, 41]. P301S mice injected with tauopathic seeds show tau proliferation into regions that are spatially distal but well connected to the seed region [2, 4, 19, 20]. Mice transgenic for only wildtype human tau (hTau) injected with proteopathic seeds show tau progression into areas heavily connected with the inoculated region [9].

However, whether progression of tau inclusions is primarily driven by anatomic connectivity with affected regions is controversial. Some authors assert regional tau deposition is a matter of neuronal-subtype dependent mechanisms [43]. Gene expression alterations can occur in “early Braak stages or when only a few NPs can be detected [indicating] that multiple neurobiological systems must be affected and engaged before...neuropathological lesions become manifest” [14]. Due to the existence of gene expression differences between brain areas, some hypothesize, without considering spread due to cell-extrinsic factors such as anatomic connectivity, “*that large-scale regional vulnerabilities in AD are likely due to the many small differences in gene expression patterns between brain regions*” [28].

A more precise argument in favor of cell-intrinsic and regional gene expression based hypotheses for explaining regional vulnerability is that upstream regulators of tau pathophysiology are innately arranged within the brain in a manner that explains spatiotemporal tau pathology progression. For example, regional differences in neuronal subtype composition, as measured by gene expression profile or morphological comparison, are posited to underwrite selective regional vulnerability to protein pathology, as certain pyramidal neuron subtypes appear particularly vulnerable to tau inclusions [13, 18, 37, 38]. Lower regional expression of MAPT in the cerebellum, as compared with the rest of the brain, is hypothesized to underlie cerebellar resistance to degeneration in tauopathic disorders such as AD [8], while higher local expression of pro-aggregation and pro-inflammatory factors corresponds with the likelihood of observing tau pathology in a given region [11, 12].

We accordingly undertake the present study to disentangle whether connectivity or regional gene expression plays a more critical role in tau proliferation patterns. We first examine mouse experiments with exogenously seeded tau to demonstrate that connectivity to seed region predicts subsequent regional tau deposition better than does a given region’s gene expression profile similarity with the seed region. We next examine whether higher expression across genes that promote tau aggregation and those that promote MAPT expression, as well as genes related to noradrenergic neurotransmission, have the same predictive power as connectivity with the seed region in determining tau progression. We find that connectivity outperforms regional expression of these known risk factors. We next employ the Network Diffusion (ND) model [33], a graph theoretic model of proteinopathy transmission over time, on the mesoscale mouse connectome [10]. We find that transmission based on the mouse brain’s connectivity network outperforms a model transmitting pathology based on regional gene expression profile similarity in recapitulating empirical tau progression in transgenic mice.

These results address an open question in the field, as some studies assert that even when tau pathology is exogenously seeded, cell-intrinsic factors might still be primary drivers of regional pathology vulnerability [24]. By analyzing exogenously seeded mouse data we were able to establish the cell-extrinsic basis of pathology progression. Interestingly, the same conclusion was reached on non-exogenously seeded transgenic mice, indicating that tau progression is driven by connectivity rather than by regional gene expression profiles, regardless of exogenous or endogenous pathology initiation. However, we here include an important caveat that region gene expression was much more predictive of regional tau pathology in non-seeded as compared with seeded mouse models.

## Methods

### Study selection

The datasets of spatiotemporal mouse pathology used in the present research were chosen based on the following criteria: a study had to characterize tau pathology in at least 10 distinct brain regions across at least 2 timepoints spanning a total period of at least 6 months. The average number of regions quantified across studies was 89 areas; specific numbers of regions quantified per study are discussed below in this subsection. Every study used mice with a C57/BL6 background so that mouse strain effects did not confound the results. All mice possessing a tau mutation had to have the same transgene background and so were all PS19 mice possessing the P301S familial FTD derived tau mutation, with mutant human tau transgene expression driven by the same promoter (MoPrP). One dataset used mice possessing both tauopathic and amyloidopathic transgenes, created by crossing the P301S tau transgene mice as above with mice possessing the human APPswe transgene [17]. In 5 of the 6 datasets cited in the present research mice were injected at between 2 and 3 months of age with a pathogenic tau infusate; specific injectate details differ from study to study, but included brain homogenate from Down Syndrome and AD (102 regions quantified) as well as CBD (96 regions quantified) tauopathy patients [4] as well as mouse models ([9]; 11 regions quantified), purified mutant tau [19], and synthetic short chain fibrils produced using cDNA cloning in *E. coli* vectors [19] injected into the hippocampus (148 regions quantified) and striatum (132 regions quantified). The study using PS19 x APPswe mice did not have exogenous seeding of pathology but still characterized the spatial development of tau pathology ([17]; 45 regions quantified). All studies used semi-quantitative, regionally realized tau pathology grading as their regional pathology measurements. Specific methodological information on each study can be found in the relevant citations, which are all listed above.

### Connectivity networks

Connectivity data was taken from the supplementary dataset published along with the mesoscale mouse connectome from the ABI (MBCA; [29]). Total projection volume between regions was generated by multiplying element-wise by the rows the connectivity matrix times the number of voxels in each seeding region. As the MBCA connectivity matrix, retrieved from SI.4 in [29], gave per-voxel normalized connectivity strength, our adjustment of connectivity to the size of each region approximates total connectivity; this procedure for approximating total axon volume or connectivity between regions is laid out in detail in SI.5 in [29]. We then averaged the resulting directed connectivity matrix, *N*_*C*_, with its transpose,${N}_C^T$, to get the standard undirected connectivity matrix used in prior graph theoretic neuroscience models [7], including the network diffusion model [33]. We employed undirected networks rather than directed networks because recent studies on transsynaptic tau spread indicate that directional transmission biases remain ambiguous [41]. Following this, we applied a thresholding criteria of getting rid of all values that were less than 0.05 the standard deviation of the nonzero entries of *N*_*C*_, resulting in a network density < 0.14. The resultant network was a sparse matrix of 426 × 426 regions, with each cell representing thresholded approximate total axon volume.

### Genetic proximity networks

In the present research, we created 3 distinct genetic proximity networks: the first network used characterized the interregional genetic expression profile similarity across all 4500 genes in the Coronal Mouse Gene Expression Atlas from the Allen Institute [26]. The second and third characterized the similarity in expression profile across smaller subsets of genes; the subsets were genes known to exert profound effects on misfolded tau aggregation and tau gene expression [12], as well as genes necessary for the synthesis and degradation and the receptors of norepinephrine, the monoamine neurotransmitter theorized to be an important factor in tauopathic disease genesis [27]. Full lists of genes from the tau pathology related and noradrenergic related specific gene expression profile networks can be found in Additional file 1: Table S1.

Interregional networks of gene expression profile similarity or proximity were calculated using the following method: First, a gene expression profile discrepancy matrix, *D*, was created, where each entry in the matrix was an integer corresponding to the number of genes, between any two regions, that were more than 3-fold differentially expressed, a methodology previously validated by the Allen Institute [15]. This discrepancy matrix was then inverted and exponentiated to create a proximity network with values normalized to a range from 0 to 1, using the following equation:

1 $$ {N}_G={e}^{-D/\lambda } $$

In Eq. (1) above *N*_*G*_represents the resulting gene expression proximity network, *D* represents the original discrepancy matrix, and *λ* is the mean of nonzero values from the discrepancy matrix, used above for normalization of the resulting values. *N*_*G*_was then thresholded to boost the signal of regionally similar gene expression profiles above the noise of the endogenously dense matrix; any values below the mean plus one standard deviation of nonzero and non-1 values was set to 0, signifying that for our purposes these two regions had effectively maximally dissimilar gene expression profiles, and resulting in a network density < 0.3, still more dense than that of *C*. *N*_*G*_will refer to the genetic expression network calculated across the entire set of sequenced genes in the MGA, *N*_*T*_will refer to the network calculated only on the set of tau expression and aggregation related genes, and *N*_*N*_will refer to the gene proximity network calculated only for noradrenergic neurotransmitter related genes.

### Regional gene expression

In addition to the gene proximity networks, we also examine whether higher regional levels of MAPT and tau aggregation related genes, as well as higher expression levels of noradrenergic related genes, relate to regional tau pathology severity. To get measures of regional gene expression levels we extracted the normalized gene expression intensity from the Allen Institute MGA for our genes of interest and summed the expression intensity across those specific genes. This resulted in a vector that had one measurement of gene intensity per region from the connectivity and genetic atlases for our genes of interest, for each of the two aforementioned gene groups. In all cases out suite of specific gene sets, across tau aggregation related [12], tau transcription promoting [3], and noradrenergic neurotransmission related genes [27], were derived from prior work. A complete list of genes used can be found in Additional file 1: Table S1. Among genes listed in [12], we selected those that are either general proteinopathic aggregation risk factors or specifically tau related, and excluded any genes that are solely related to amyloid beta. We included PrP in the list of tau expression promoting genes because this was the promoter sequence used to drive tau transgene expression in all analyzed mouse datasets.

### Spatial diffusion modeling as an alternative model to graph diffusion

We additionally created a spatial diffusion model as a comparison or alternative hypothesis to the graph diffusion model. The spatial diffusion model was based on the same fundamental network diffusion eq. (2) stated below. The difference between ND and spatial diffusion in the present study is that the network for spatial diffusion is a matrix where each entry in the matrix *N*_*D*_(*i*, *j*)is the reciprocal of the Cartesian distance between the center of mass of each GM region included in the Allen Institute’s mouse connectivity atlas. Using this distance matrix, *N*_*D*_, rather than the connectivity matrix *N*_*C*_, we ran the diffusion equation stated above in [2] to get a model approximating diffusion based on spatial proximity, which will be referred to as SPD.

### Seed region proximity analyses

**Fig. 1:**
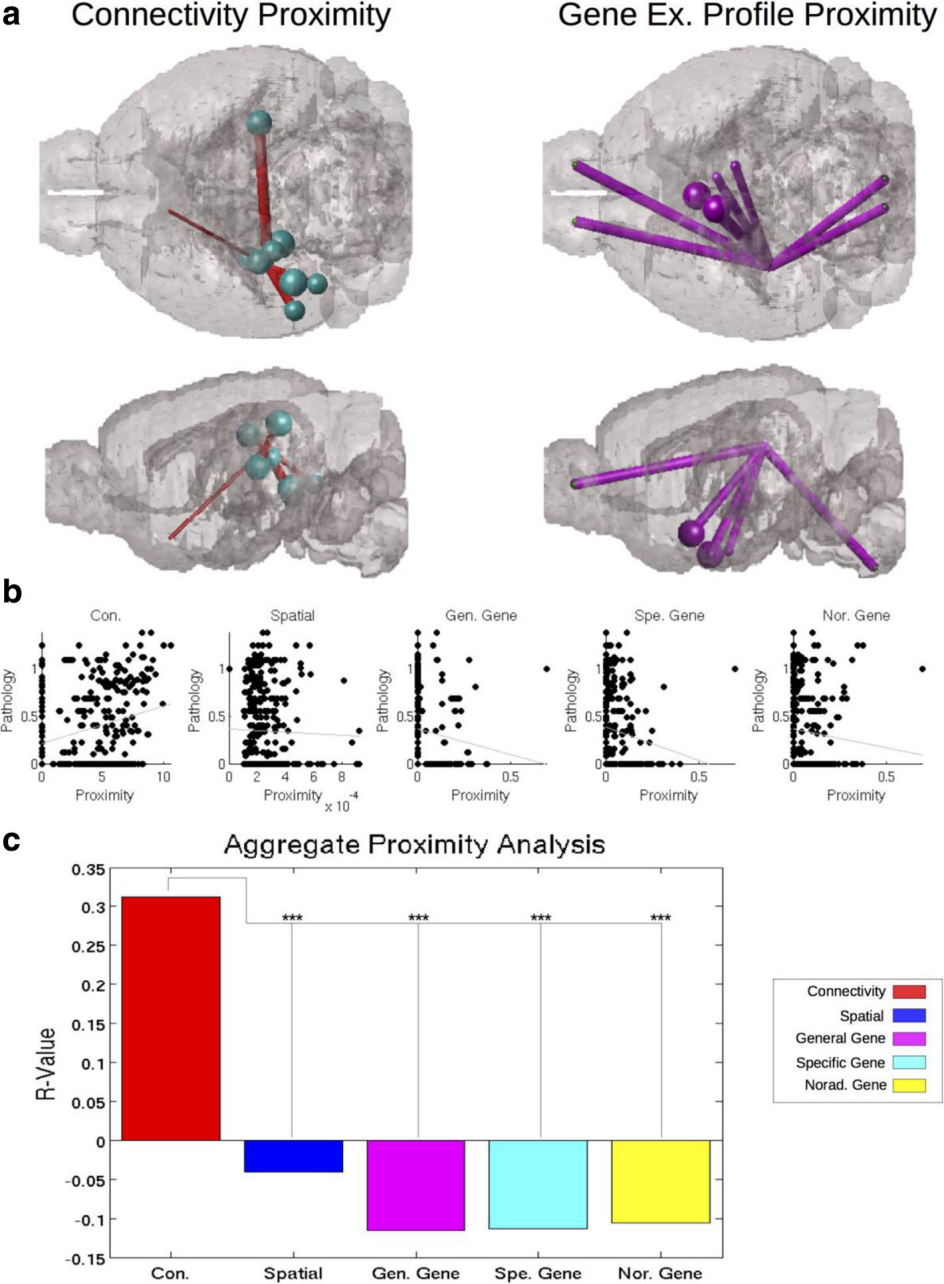
Connectivity proximity better correlates with regional pathology severity than gene expression profile proximity. Here proximity is demonstrated in terms of connectivity and gene expression profile, using the 10 regions most proximal to the CA1 seed region from [4]. The thickness of each pipe represents how proximal each region is with CA1, with thicker pipes indicating higher proximity, while each ball represents the regional tau pathology severity. **a** Connectivity proximity with CA1 corresponds better with regional tau proteinopathy severity than does (**b**) gene expression profile proximity with CA1. In an aggregated meta-dataset of all exogenously seeded mouse studies used in the present work, connectivity produced the best fit with empirical regional tau pathology data (**b**) and produced the best, only positive, and significantly strongest relationship, as measured by r-value and tested with Fisher’s R-to-Z Test, with regional tau pathology data (**c**). *** *p* < 0.001, in the Fisher’s R-to-Z Test for comparing r-values

For the 5/6 datasets that had reported seed regions we performed what we term a Proximity Analysis. An example of this can be seen in Fig. 1. These analyses involved calculating the average connectivity, spatial distance, or genetic similarity with a given seed site or sites on a region by region basis. This produced a 426 × 1 vector, corresponding to 426 Gy matter regions available in the mouse atlas. We then compared this vector with that of empirical regional pathology from each study and an aggregated meta-dataset using a natural log transformed regression, as proximity data in all networks as well as empirical data were exponentially distributed and would give erroneously high r-values due to outliers with standard linear regression. We created the aggregated meta dataset by vertically concatenating each the data from each dataset in the y-vector, and each dataset’s corresponding predictor vector in the x-vector. As datasets were measured on different scales, the values in the y-vector were normalized by division by the maximum value, on a per dataset basis. We then performed a natural log transformed regression as above.

### Network diffusion

A previous graph theoretic model of pathology progression in AD throughout a brain network was shown to be predictive of future patterns of disease progression [33]. The model captures the diffusion of the disease factor throughout the network via the Network Diffusion equation:

2 $$ {X}_{GD}(t)={e}^{\left(-\beta Lt\right)}\cdotp X(0) $$

This models the long range patterns of progression of the protein pathology at any time *t* as a product of the initial seeding pattern *X(0)*, and the so-called diffusion kernel *exp.(−βtL)*, with diffusion and time constants, *β* and *t*, [33], and the network Laplacian matrix, *L*. The Laplacian is defined as [33, 34]:

3 $$ \mathrm{L}=\mathrm{I}\hbox{--} {\mathrm{D}}^{\left(-1/2\right)}\cdotp \mathrm{N}\cdotp {\mathrm{D}}^{\left(-1/2\right)} $$

where *N* is the 426 × 426 connectivity matrix giving the strength of connections between all region pairs. Since we are interested in understanding how the same canonical network diffusion model gives pathology progression using various proximity networks, we therefore defined separate 426 × 426 matrices corresponding to pairwise proximity determined, respectively, using tracer-based connectivity, spatial distance, and gene expression similarity networks. These are denoted respectively by matrices *N*_*C*_ , *N*_*D*_ , *N*_*G*_ , *N*_*T*_ , *N*_*N*_. Note that we defined 3 different gene-based similarity matrices *N*_*G*_ , *N*_*T*_ , *N*_*N*_, corresponding to general, tau-specific and noradrenergic gene expression, respectively. For each proximity matrix, the corresponding Laplacian was defined using Eq. (3).

The major difference with previous ND model is that because we are interested in total pathology accumulation over time, we model tau progression as a summative or iterative process:

4 $$ {X}_{NT}(i)={e}^{\left(-\beta Lt\right)}\cdotp X\left(i- 1\right)+X\left(i- 1\right) $$

We use eq. (4) to calculate, for any point in time, the deposition of tau across the brain regions represented in our connectivity, spatial distance, and gene expression networks. Further information on the original network diffusion equation and its mathematical foundation can be found in both [1, 33]. The symbol meanings in Eq. (4) are the same as in Eq. (2).

The result from the network diffusion equation was, akin to proximity analyses, a vector with one entry per region represented in the connectivity, spatial distance, and gene expression networks. However, the ND model produces predictions of regional pathology, not a simple empirical measurement of network proximity with a seed region, and so does not require a seed region, but only a baseline pathology measurement. Akin to the proximity analyses discussed above, we compared our prediction vector from the ND model, run with the *L* from each network, to the regional pathology measurements from each dataset using a natural log transformed regression. We used both baseline measurements and, where available, reported seedpoints, as the initiation point for the ND model. An example of the ND model and how to interpret its results can be found in Fig. 3. Note in particular Fig. 3b: here we show both how we calculate *βt-*values, by setting *β* = 0 and modulating *t* to the value that produces the strongest correlation with the data, and how we assess predictive value added, by calculating the change in r-value from baseline to peak *βt*-value, in this manuscript referred to as Δr.

### Comparing predictive value across different predictors

When comparing r-values, *p*-values, and fits across predictions from proximity or ND modeling using any of the connectivity, gene expression profile, or spatial distance networks, we employed two methods. First, using separate bivariate analyses, we obtained Pearson’s r-values between regional tau and either connectivity or gene expression. We compared the resulting r statistic directly using Fisher’s R-to-Z Test, and obtained a *p*-value for the likelihood of a true difference between r-values associated with different predictors. Next, we used a Multivariate Linear Model, and entered predictions from connectivity networks, regional gene expression across tau aggregation and transcription related, as well as noradrenergic related, genes, and seed region or baseline regional pathology data, as separate predictors. From this we could calculate independent per-predictor r and *p*-values, which we used as the basis of our comparisons. All analyses were performed using the following methods for creating the prediction and data vectors: we used only the sampled regions from each dataset in our regressions and multivariate linear models, and 2) we used all 426 regions from the MBA, with 0 pathology given in each region that went unmeasured in our y-variable vector. All above statistics were performed in MatLab.

## Results

### Assessing connectivity versus gene expression profile similarity with seed regions via bivariate correlation

To quantify the role connectivity and gene expression profile play in tau progression from a seed region we first tested whether higher anatomical connectivity with a seed region or degree of gene expression profile similarity with a seed region was a better predictor of that region’s tau pathology severity measured at the end of the study, as well as of the longitudinal slope of measured tau over the duration of the study. An anatomical example of what is meant by connectivity and gene expression profile similarity can be found in Fig. 1a. From this panel, we demonstrate that connectivity proximity with the seed region (in this case, CA1) was a better determinant of regional tau pathology severity than was gene expression profile proximity with the seed region. Note that regions most heavily connected with the seed region (here CA1) uniformly exhibit more tau pathology at post-injection time points, whereas the regions most genetically similar to the seed location do not, consistent with the results of our across datasets analyses.

**Fig. 2:**
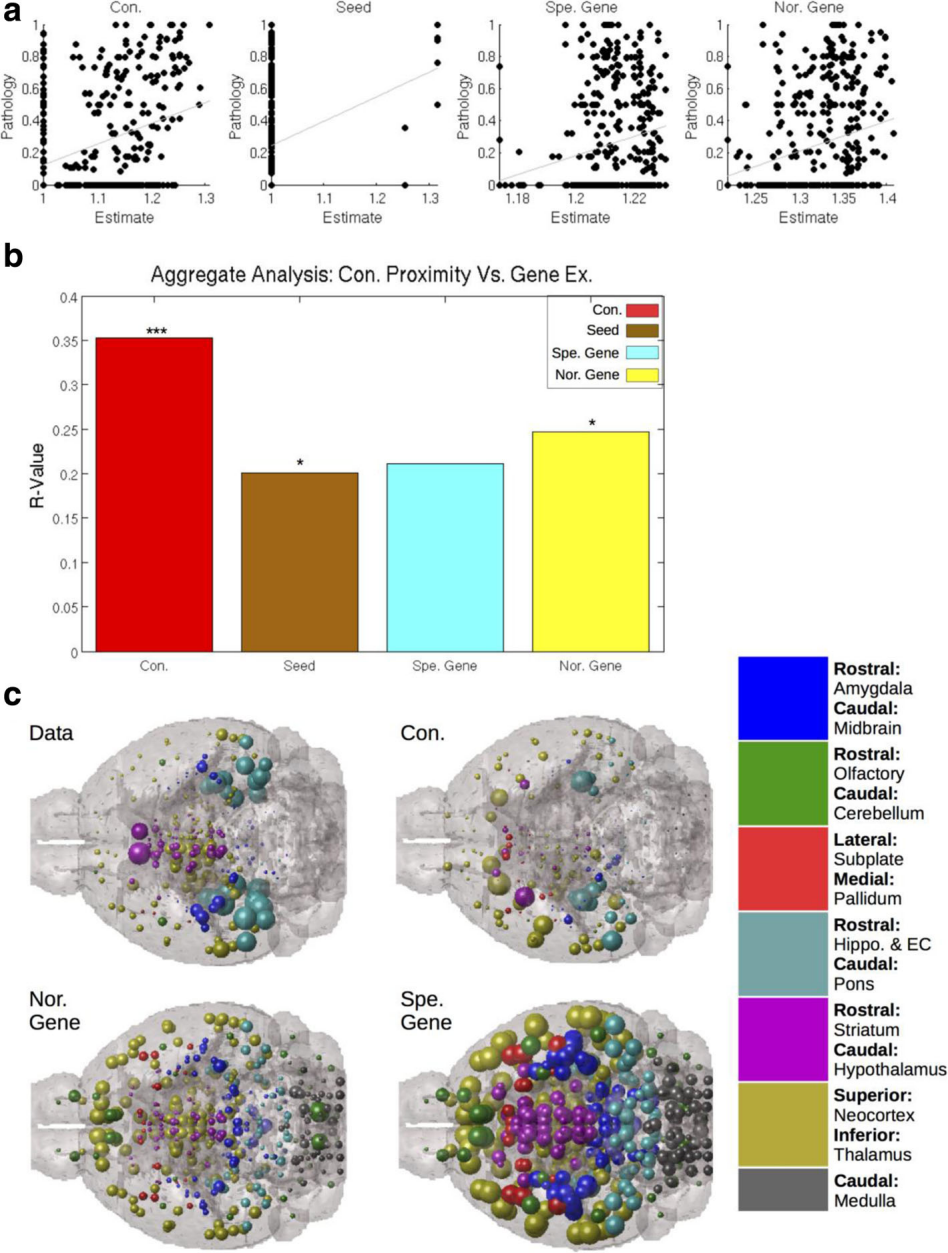
Connectivity with the seed region better predicts regional tau pathology than does the regional expression level of tau aggregation and transcription promoting and noradrenergic neurotransmission related genes. **a** Connectivity with the seed region produced the best correlate with regional tau pathology severity data in seeded datasets. **b** A Multivariate Linear Model factoring out the individual contributions of connectivity proximity, seed region location, and the regional gene expression levels towards predicting regional tauopathic severity indicates that connectivity with the seed region is the most significant predictor, as indicated by the *p*-value threshold indicators within the bar chart. **c** Anatomic illustrations illustrate the above statistical analyses. Larger spheres correspond with more predicted pathology by each modeled, as labeled. The color and anatomic location legend to the right of the anatomic illustrations gives the sphere color and location corresponding to each major region in the brain. This color and location scheme is replicated throughout all anatomic illustrations in this paper. *** *p* < 0.001, ** *p* < 0.01, * *p* < 0.05

Across all five datasets citing exogenous seeding, apart from one (“Boluda CBD”; [4]), connectivity with seed regions was a better predictor of post-injection regional tau pathology severity than was similarity in gene expression profile to seed, or spatial distance from seed (Table 1; Fig. 1a-b). Since no single study reported all possible affected regions, we repeated this analysis on a meta-dataset created by aggregating all five studies into one (called “Aggregated meta-dataset”, right column in Table 1). On this meta-dataset, connectivity with the seed region was the only significant predictor of regional tau pathology levels at the last measured timepoint of the study, *r* = 0.35, *p* < 0.001. None of the ways in which we measured similarity in gene expression to seed, whether across all sequenced genes (“General gene expression”), or across a suite of genes known to promote tau aggregation and expression (“Specific Gene Expression”), or across the group of noradrenergic neurotransmission related genes, were significantly correlated with regional proteinopathy. Scatter plots showing these correlations against the metadataset are in Fig. 2a. Fisher’s R-to-Z test on these r-values yielded that regional connectivity with seed is significantly better at predicating regional tau pathology severity, compared to gene expression, or spatial distance, *p* < 0.001 (Fig. 1b). All scatterplots for per study analyses can be found in Additional file 2: Figure S1 and all r-values can be found in Table 1.

**Table 1:** This table presents all of the r-values and *p*-value thresholds reported in Figs. 2 & 3 and in the corresponding parts of the Results section

	Bolunda DSAD	Bolunda CBD	Iba Hipp. Inj.	Iba Str. Inj.	Clavaguera	Aggregate Meta-Dataset
Mouse Model	P301S	P301S	P301S	P301S	Alz17	/
Infusate	DSAD Homogenate	CBD Homogenate	Synthetic Tau Fibrils	Synthetic Tau Fibrils	P301S purified tau fibrils	/
Seed Region	CA1 & V1	CA1	CA1 & CA3	Caudoputamen	Hippocampus	Aggregate Meta-Seed
PROXIMITY (BIVARIATE CORRELATIONS)						
Connectivity Proximity	0.34*	0.20	0.56***	0.37**	0.64**	0.35***
Spatial Proximity	0.05	−0.14	−0.02	−0.23*	0.17	−0.04
General Gene Proximity	−0.01	−0.10	−0.06	−0.38**	0.04	−0.12
Specific Tau Gene Proximity	0.05	0.29*	−0.08	−0.50***	0.05	−0.11
Noradrenergic Gene Proximity	−0.24*	−0.18	0.04	−0.28*	−0.18	−0.10
ABSOLUTE REGIONAL GENE EXPRESSION VS. CONNECTIVITY (MULTIVARIATE LINEAR MODEL)						
Connectivity Proximity	0.35***	0.20	0.56***	0.38*	0.65**	0.35***
Summed Specific Tau Gene Ex.	−0.14	0.04	0.21	0.46**	−0.34*	0.21
Summed Noradrenergic Gene Ex.	0.28*	0.20	0.23	0.41*	0.56*	0.24*

The aggregated and per dataset r-values are all reported in this table. The r-values in each dark gray row separated section of the table were obtained independently from a standard Pearson Correlation to be compared with other proximity measurements (the top value) and from a Multivariate Linear Fit Model (the bottom value). Values above the dark row titled Lin. Mod. are using regional proximity to the seed region as the predictor of tau pathology severity, whereas values below that row compare connectivity proximity and regional gene expression, calculated using the Multivariate Linear Fit Model. *** *p* < 0.001, ** *p* < 0.01, * *p* < 0.05

### Comparing connectivity proximity with seed regions with absolute regional gene expression levels using a multivariate linear model

While *similarity* with the seed’s gene expression profile was not predictive of regional tau, we hypothesized that higher *absolute* expression of tau- or noradrenergic-related genes might predict regional tau better than connectivity with the seed region. To test this, we built a multivariate linear model whose outcome was regional tau at the final regionally quantified timepoint post-seeding, and whose predictors were connectivity-to-seed, and both types of gene expression profiles. We included the seed region’s tau severity as an additional predictor, since the seeded region continues to display elevated levels of tau over time. In this model, we did not include the general gene expression profile from all genes, as it was found to be a poor predictor in the previous analysis. We found that connectivity consistently, across datasets, explains observed tau pathology patterns than does regional gene expression profile. The same was true on the aggregated meta dataset, *r* = 0.35, *p* < 0.001 (Table 1, right column; Fig. 2a-b). Seed region and noradrenergic related gene expression levels were found to correlate with regional tau pathology severity, *r* = 0.20 and *r* = 0.24, respectively, *p* < 0.05 (Table 1; Fig. 2a-b), but not as significantly or strongly as connectivity with the seed region. Neither tau nor noradrenergic related gene regional gene expression levels correlated with regional tau pathology severity. In exogenously seeded mouse models, connectivity with the seed region was found to be the best predictor of regional pathology severity. Anatomic illustrations can be found in Fig. 2c. Scatterplots for individual study by regional gene expression analyses can be found in Additional file 2: Figure S2.

### Assessing the predictive power of network diffusion modeling using connectivity and gene expression profile similarity network using bivariate correlations

The above results show that the proximal spread from the exogenous seed is explained better by connectivity to seed compared to gene expression. We now wish to test whether ongoing pathology progression is also primarily driven by connectivity or gene similarity between all possible brain regions, beyond just proximity or similarity with the seed region. This includes the possibility that pathology could spread widely across the brain, into further regions unrelated to the seed. For this purpose, we lean on the mathematical Network Diffusion (ND) model that was previously shown to recapitulate ongoing pathology transmission in human anatomic brain networks [33]. The ND model allows us to test hypotheses regarding pathology spread on both anatomic connectivity networks, as well as progression that depends on inter-regional similarity in gene expression, assuming that tau pathology is being driven to take hold in regions genetically most similar to those already exhibiting tau pathology. Of note, when we use the terminology longitudinal or slope, we are not referring to repeated measurements within the same animal, but rather the pathology progression observed in the group tau pathology patterns of mice sacrificed at different timepoints post-injection or post-birth.

**Fig. 3:**
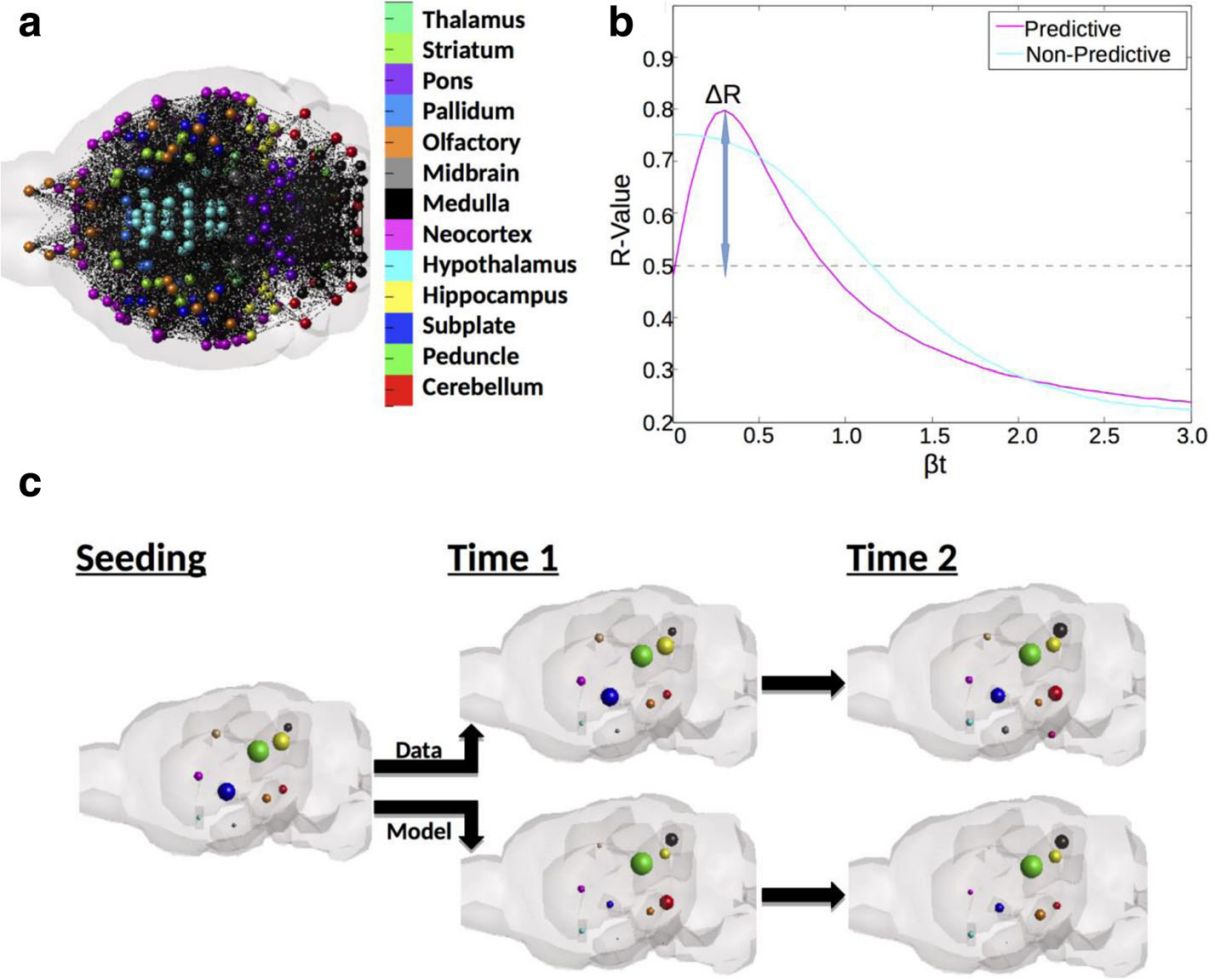
An example of ND modeling and associated analyses. **a** Here we demonstrate what is meant by representing the brain as a network, with each ball indicating the center of mass of a region and each line between any pair of regions implying the presence of a connection between them. **b** Here we demonstrate the parameter optimization or βt curve for ND modeling. A predictive curve, or one where the network used for ND modeling adds predictive value beyond a starting point has a defined peak at βt > 0, whereas a non-predictive model will show a peak at βt = 0. The delta-R measure indicated here refers to the increase in r-value from baseline given by ND. **c** Here we provide an anatomic illustration of the predictions over time given by ND modeling about regional pathology severity, with each ball representing a region, and the size of the ball indicating the degree of pathology severity

We created 4 networks, defined mathematically by 426 × 426 matrices *N*_*C*_ , *N*_*D*_ , *N*_*G*_ , *N*_*T*_ , *N*_*N*_ (see Methods), representing inter-regional connectivity, and 3 kinds of gene expression similarity, respectively. The connectivity graph *N*_*C*_is shown in Fig. 3a, and similar ones for the gene expression similarity graphs can be envisaged. On each such graph, we applied the ND model, using Eq. (4), to produce predictions of future pathology progression *X*(*t*), starting from the seed configuration *X*(0)which is defined as a zero vector with 1 at the seed location. At each time point t, the *X*(t) vector is compared with empirical regional tau data via Pearson correlation, to produce*r*(*t*). Examples of the resulting r(t)-curve are shown in Fig. 3b, which shows how well the ND model matches empirical data as initial pathology *X*(0)diffuses into the rest of the network. The improvement over the match (r-value) with the seed configuration *X*(0) (indicated as *Δr*) is the most important metric for assessing the predicitive value of the model, and is reported in Table 2, using both connectivity and gene expression networks. The ND model can be considered indicative of ongoing network progression only if *Δr* > 0. An example anatomic illustration of the predictions the ND model over time is depicted in Fig. 3c.

**Table 2:** ND modeling to assess the relative contribution of connectivity and gene expression profile in explaining tau progression and distribution

	Measure	Boluda DSAD (6 Mo)	Boluda CBD (6 Mo)	Iba Hipp. Inj. (6 Mo)	Iba Str. Inj. (6 Mo)	Clavaguera (15 Mo)
Mouse Model	/	P301S	P301S	P301S	P301S	Alz17
Infusate	/	DSAD Homogenate	CBD Homogenate	Synthetic Tau Fibrils	Synthetic Tau Fibrils	P301S Purified Tau
Seed Region	/	CA1 & V1	CA1	CA1 & CA3	Caudoput.	Hippocampus
ND USING STUDY SELECTED REGIONS (BIVARIATE CORRELATIONS)						
Connectivity, Deposition	ΔR	0.30	0.22	0.45	0.23	0.25
Connectivity, Slope	ΔR	0.27	0.23	0.40	0.11	0.47
Spatial, Deposition	ΔR	0.01	0.00	0.01	0.00	0.03
Spatial, Slope	ΔR	0.02	0.00	0.01	0.00	0.01
General Gene, Dep.	ΔR	0.08	0.00	0.10	0.10	0.01
General Gene, Slope	ΔR	0.17	0.01	0.28	0.08	0.22
Specific Gene, Dep.	ΔR	0.01	0.02	0.03	0.02	0.00
Specific Gene, Slope	ΔR	0.02	0.02	0.22	0.00	0.05
Noradren. Gene, Dep.	ΔR	0.00	0.00	0.00	0.07	0.00
Noradren. Gene, Slope	ΔR	0.00	0.00	0.01	0.03	0.00
ND-CONNECTIVITY VS REGIONAL GENE EXPRESSION (MULTIVARIATE LINEAR MODEL)						
Connectivity	T-Stat	12.74***	8.97***	10.51***	2.90**	2.50*
Seed or Baseline	T-Stat	−0.31	1.40	0.28	0.50	1.69
Summed Specific Gene Ex.	T-Stat	−1.85	−2.05*	−2.42*	2.53*	0.32
Summed Noradren. Gene Ex.	T-Stat	2.64**	1.67	1.92	0.44	0.19

The entries below the “Bivariate Correlations” row correspond to the ΔR obtained from running the ND model with each row’s network from reported seedpoint. The four entries after the “Multivariate Linear Model” row represent the t-values and *p*-value thresholds obtained from ND model predictions or summed regional expression predictions after they were input as independent predictors into a Multivariate Linear Fit Model. *** *p* < 0.001, ** *p* < 0.01, * *p* < 0.05

**Fig. 4:**
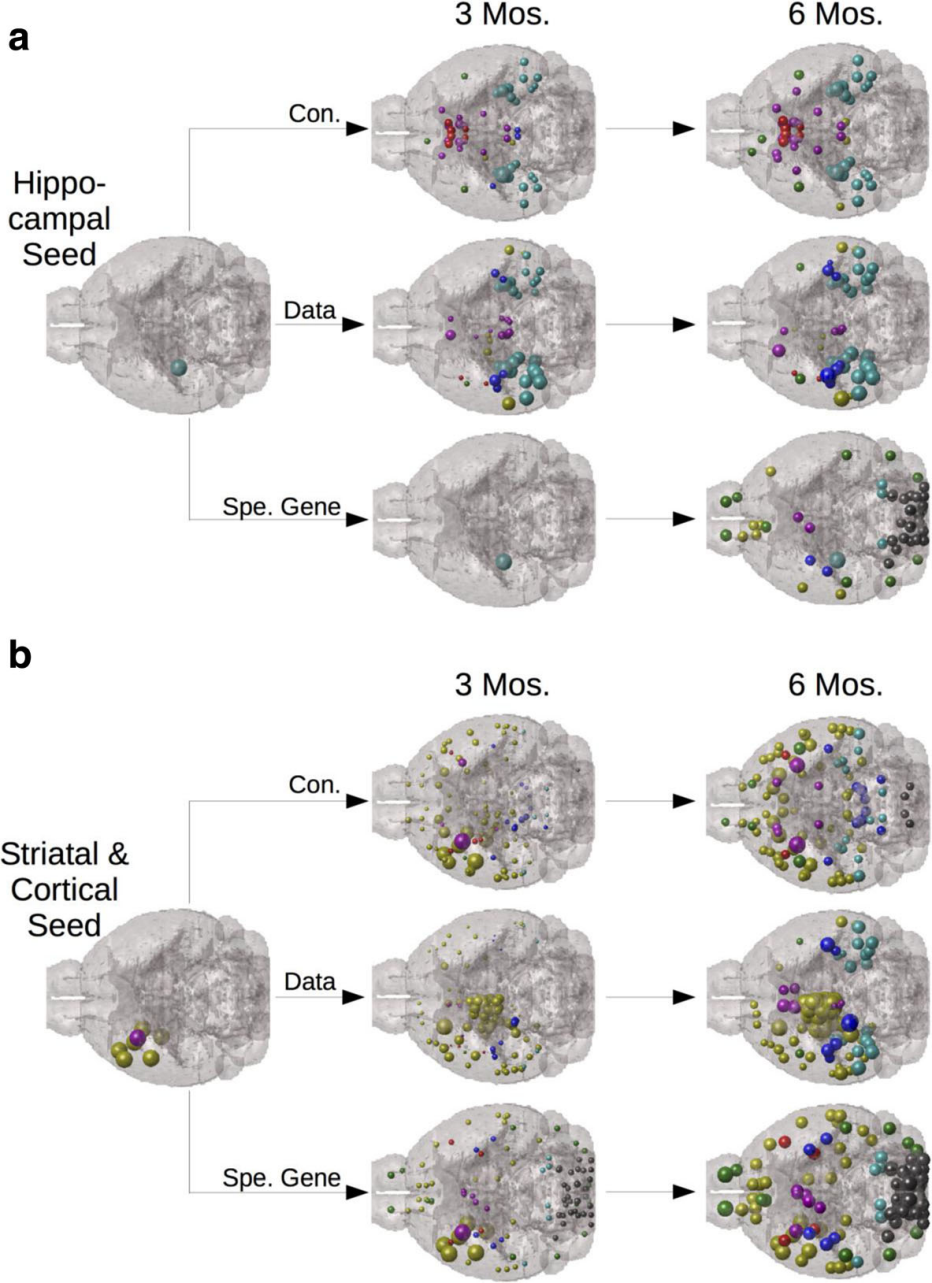
ND modeling from seedpoints using the connectivity network best fits empirical spatiotemporal tau pathology progression in a seed independent manner. **a** Regardless of whether seeding occurs in the hippocampus or (**b**) the striatum and overlying neocortex, ND modeling using the connectivity network better fits empirical data than does ND modeling using any other network, across all timepoints. Here we use ND modeling with the specific tau aggregation and transcription promoting gene expression profile similarity network as our comparison. The two datasets, using different seed locations, both come from [19]. The color and location legend for this figure is the same as in Fig. 2, and once again, sphere size corresponds with degree of predicted pathology in a given area

Across all exogenously seeded studies, ND using the anatomic connectivity network was a significant predictor of regional tau pathology severity patterns, as well as regional slope of tau pathology increase from empirical seedpoint; this was not true for progression based on genetic expression profile or spatial proximity networks using ND (Table 2). In all seeded studies, whether seeded in the hippocampus, striatum, or neorcortex, ND using the connectivity rather than gene expression profile similarity networks, best recapitulated the spatiotemporal pattern of tau pathology (Table 2; [19]). As illustrated in Fig. 4a-b ND using connectivity network is the best model for predicting tau pathology from an exogenous seedpoint in a seed location independent manner. In the above regressions, we included only those regions which were reported to have an empirical “signal”, meaning those regions for which the study in question reported a tau value, zero or otherwise. We further tested whether our results were largely driven by the present datasets’ regions selected for quantification, by including all 426 regions in our analyses, regardless of whether they were reported by the empirical study or not. Using this retesting methodology, we again found that as measured by *Δr*, across all studies, ND using the anatomic connectivity network best recreated empirical pathology patterns (Additional file 1: Table S2; top section). ND modeling proceeding from reported seedpoint against all five exogenously seeded studies can be found in Additional file 2: Figure S3 for ND using only study-selected regions, and in Additional file 2: Figure S4 for ND using all 426 ABA (Allen Brain Atlas) regions.

### Comparing ND using the connectivity network with absolute regional gene expression levels via a multivariate linear model

Given that ND model based on gene expression similarity networks was not a significant predictor, we next tested whether absolute regional expression profile was by itself a significant predictor instead. The results are contained in Table 2 (bottom section), and show the t- and p-statistic of each predictor in the linear model, for each exogenously seeded mouse study. We found that ND model based on connectivity outperformed absolute gene expression across all seeded studies. Furthermore, ND using the connectivity network was the only consistently significant predictor, across all seeded studies, of regional tau pathology severity.

The overall conclusion from Table 2 and Additional file 1: Table S2 is that ND using the anatomic connectivity network was the best model for predicting the patterns of regional tau pathology severity, outperforming both ND using gene expression profile similarity networks and absolute regional gene expression of known tau aggregation related genes, as well as noradrenergic neurotransmission related genes. We found this in all exogenously seeded studies, for both tau deposition at the last measured timepoint and its longitudinal slope, and regardless of whether we analyzed modeled results of regional tau pathology using only the study selected regions (Table 2) or using all 426 ABA regions (Additional file 1: Table S2).

### Modeling tau pathology using connectivity and gene expression profile in a non-seeded mouse dataset

**Fig. 5:**
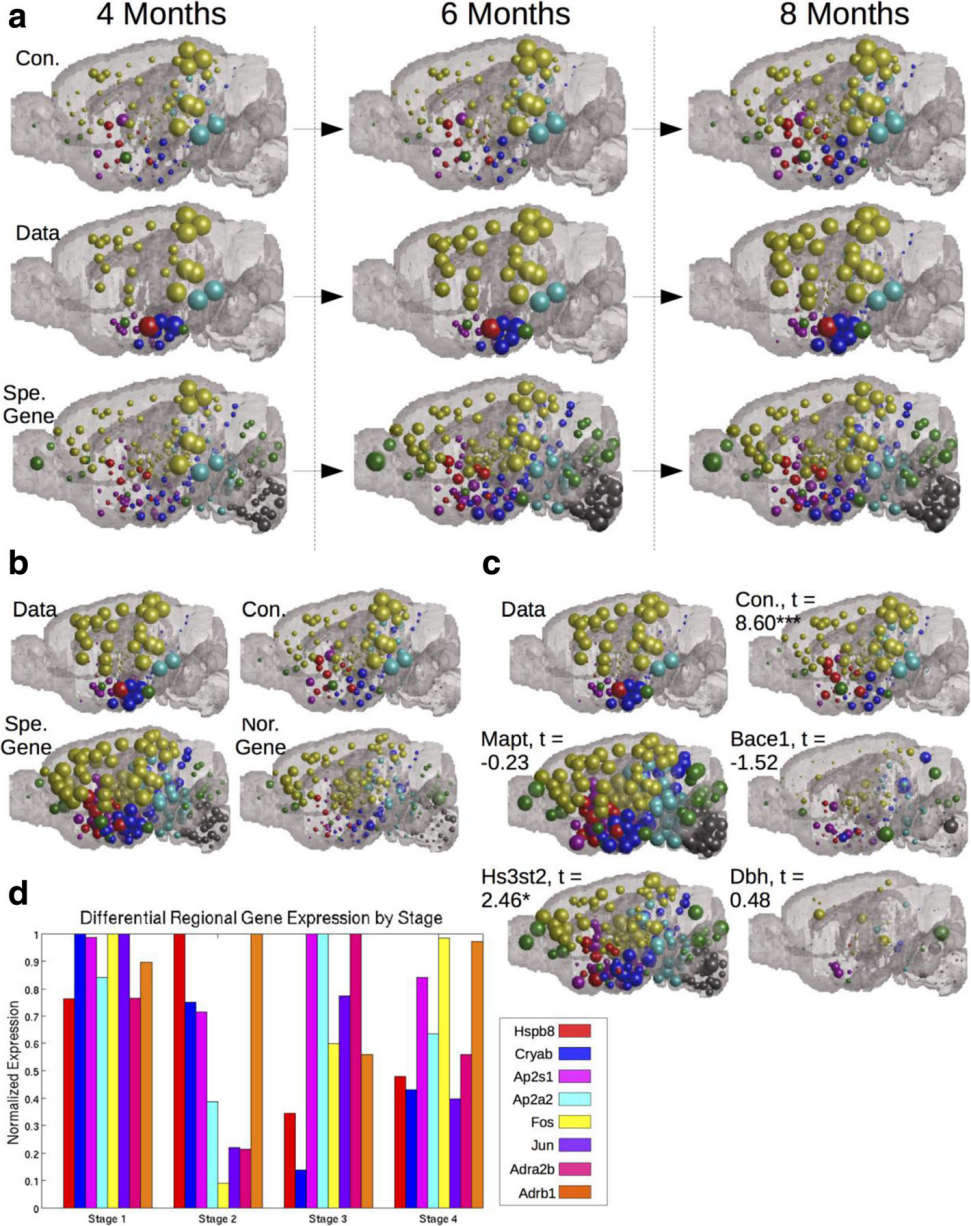
ND modeling indicates connectivity is a better predictor of tau pathology progression and regional vulnerability than regional gene expression but that regional gene expression does better in the non-seeded mouse dataset than in seeded datasets. **a** An anatomic spatiotemporal illustration of the predictions of ND modeling using both the anatomic connectivity network and the gene expression similarity network, across a specific subset of genes known to be important for promoting tau aggregation and transcription, as compared with the empirical spatiotemporal data on tau progression patterns. **b** An anatomic illustration of the results from Tables 2 and 3—bottom-section comparing ND using the connectivity network with absolute regional gene expression (**c**) Modeling pathology using individual genes known to have a mechanistic link with proteinopathy (Mapt, Bace1, Hs3st2) or genes necessary for noradrenergic neurotransmission (Dbh) does not perform as well as ND using the anatomic connectivity network when analyzed using all 426 ABA regions. **d** Genes that are differentially expressed among regions showing baseline tau relative to the rest of the brain are not more heavily expressed in regions showing pathology at baseline or early relative to later stages. The color and location legends for the major regions in the brain illustrations are the same as in Fig. 2 and Fig. 4, and sphere size corresponds with degree of predicted pathology in a given area. * *p* < 0.05, ** *p* < 0.01, *** *p* < 0.001

Regressions using only the regions sampled by this study indicated that ND using the anatomic connectivity network, while moderately predictive, underperformed ND utilizing gene expression similarity networks across all timepoints (Table 3). However, ND using genetic similarity networks predicts widespread pathology across very many non-study-selected regions, while ND using connectivity appears to give a more sparse match with empirical data (Fig. 5a). ND using genetic similarity networks results could be artifacts of only testing a small subset of studies. To assess this issue, as above, we reran all our analyses using all 426 MBA, rather than only the areas selected for quantification in this study by [17], indicates that ND using the anatomic connectivity network is the only covariate able to significantly improve upon the baseline pathology measurements as a predictor for spatiotemporal tau progression when all 426 ABA regions are included in the analyses (Table 3). Furthermore, absolute regional gene expression of specific tau aggregation and expression promoting factors, as well as noradrenergic related genes, only recreates tau pathology as well as ND using the connectivity network when the analysis used only the study-sampled regions (Table 3). When all 426 MBA regions are used in a Multivariate Linear Model ND using the connectivity network again outperforms absolute regional gene expression (Table 3). Moreover, the anatomic match between the data and ND using the anatomic connectivity network appears superior to that between the data and regional expression of tau or noradrenergic related genes (Fig. 5b). While these present results are consistent with our findings in exogenously seeded datasets above, we were surprised that connectivity would remain the best predictor even in a completely endogenously driven transgenic mouse model. We therefore tested whether the regional expression of genes known to have a mechanistic link to protein misfolding in tauopathic disorders (Mapt; Bace1; Hs3st2) or known to be necessary for norepinephrine synthesis (Dbh) would significantly recreate end timepoint regional pattern of tau pathology. In a Multivariate Linear Model however, ND using the anatomic connectivity network remained the only significant predictor of regional patterns of tau proteinopathy severity in analyses using all 426 ABA regions; analyses using only study-selected regions yielded no significant positive results (Fig. 5c). Scatterplots and parameter optimization curves for the non-seeded mouse dataset [17] can be found in Additional file 2: Figure S5.

**Table 3:** Regression and Linear Models analyses run restricted to only study selected regions and including all 426 ABA brain regions

	Measure	4 Months Age	6 Months Age	8 Months Age
ND MODELING (BIVARIATE CORRELATIONS)				
Analysis Using Study Selected Regions Only				
Connectivity	ΔR	0.00	0.01	0.02
Spatial	ΔR	0.01	0.02	0.02
General Gene	ΔR	0.02	0.03	0.04
Specific Tau Genes	ΔR	0.08	0.13	0.12
Noradrenergic Genes	ΔR	0.05	0.10	0.10
Analysis Using All 426 ABA Regions				
Connectivity	ΔR	0.09	0.16	0.21
Spatial	ΔR	0.00	0.00	0.01
General Gene	ΔR	0.00	0.00	0.00
Specific Gene	ΔR	0.00	0.00	0.00
Noradrenergic Gene	ΔR	0.00	0.00	0.00
ND VS REGIONAL GENE EX. (MULTIVARIATE LIN. MOD.)		End Timepoint Deposition	Analysis Using Study Selected Regions Left	End Timepoint Deposition
Connectivity	T-Stat	−0.20		6.92***
Baseline Deposition	T-Stat	1.73	⇐	1.83
Summed Specific Gene Ex.	T-Stat	2.41*	Analysis Using All 426 ABA Regions Right	1.65
Summed Noradren. Gene Ex.	T-Stat	1.05	⇒	3.96***

The entries under the “Bivariate Correlations” row correspond to the ΔR obtained from running the ND model from the baseline pathology measurement as this dataset [17] had no exogenous seedpoint. The four entries after the “Multivariate Linear Model” row represent the t-values and *p*-value thresholds obtained from ND model predictions or summed regional expression predictions after they were input as independent predictors into a Multivariate Linear Fit Model. Merged row (in the bivariate analyses section) and column separators (in the multivariate linear model section) denote which statistics correspond to analyses run restricted to only study selected regions or run using all 426 ABA brain regions. For T-Stats: *** *p* < 0.001, ** *p* < 0.01, * *p* < 0.05

#### Staging

Finally, we explored whether regional gene expression could predict tau pathology staging of the endogenous mouse model. Using a t-test with a significance threshold of α = 0.05, we identified tau and noradrenergic related genes that were differentially expressed in regions showing baseline tau pathology from [17] relative to the rest of the brain. We then plotted the expression patterns of these genes across regions showing earliest pathology at the 2 months baseline stage (Stage 1), and at 4 months (Stage 2), 6 months (Stage 3), and 8 months (Stage 4) after birth to see if they were more heavily expressed in regions exhibiting pathology at earlier stages. The above definition of staging is not meant to exactly mimic the classic Braak tau stages in humans, although we aimed for a rough correspondence. We found that no pattern of regional expression of any of these differentially expressed genes predicts tau pathology staging (Fig. 5d).

## Discussion

The present study contributes to the field of neurodegenerative pathology progression in several ways. This is the first study, to our knowledge, to demonstrate transregional transsynaptic tau progression in the mouse on a macroscopic, whole brain, regionally unbiased level. Although several mouse studies have reinforced the hypothesis of trans-neuronal spread, they have hitherto been descriptive and have focused on specific regions or projections. We rigorously and quantitatively demonstrate that the brain’s anatomic connectivity network is a more important determinant of regional vulnerability and the pattern of tau pathology progression than is regional gene expression profile, both in exogenously seeded and non-seeded mouse datasets. This may therefore represent the first quantitative assessment of the relative contributions of regional gene expression and anatomic connectivity in the spatiotemporal development of tauopathic degenerative disease. That spatiotemporal tau pathology proliferation patterns might be driven mainly by anatomic connectivity is an important finding for three reasons. First, our connectivity based explanation of tau pathology proliferation argues that tau deposition is driven by architectonic or morphological properties, such as the connectivity network, rather than neuronal-subtype specific factors. Here we have considered gene expression profile as a surrogate for the molecular and cell-type signature of a brain region. Second, it argues against the hypothesis that upstream regulators of proteinopathy are innately arranged within the brain in a manner that explains spatiotemporal tau pathology progression [12]. Third, it argues against tau deposition in mice being driven by transgene specific factors, as higher regional expression of tau promoting factors do not correspond with increased tau pathology severity, but connectivity with regions already exhibiting pathology does. These novel findings in the field of tau transmission give a quantitative foundation for future studies of the spatiotemporal development of degenerative disease. These results and their implications are discussed below. However, the current study does not definitively elucidate all factors driving tau pathology transmission, as our connectivity based spread ND model does not perfectly recapitulate spatiotemporal tau development. Furthermore, it does not explore all facets related to connectivity that might be important for tau pathology transmission. For instance, it does not examine whether there is a directional predilection, going in either a presynaptic or postsynaptic direction, to tau pathology transmission. The current research should therefore be read with these caveats in mind.

### Confirming transsynaptic spread of tau pathology in mouse models on the whole brain, macroscopic scale

Tau progression data from multi-timepoint mouse studies was most accurately recapitulated with ND using *N*_*C*_ (Table 2). Furthermore, connectivity proximity with the seed region was a strong and significant predictor or regional pathology severity across all exogenously seeded mouse datasets (Table 1; Figs. 1 and 2). Unlike prior studies where a small number of regions were preselected for tau pathology quantification, our current data provide the first quantitative, regionally unbiased support to the emerging notion that hyperphosphorylated tau spreads via neural architecture, transynaptically propagating from neuron to neuron [6, 9, 16, 32]. Previous studies have focused on tau transmission across a few specific synapses [40] or a small number of regions [16, 25]. Almost all whole brain studies of pathology progression in AD have been in humans and have not tracked proteinopathy directly, but rather followed gross atrophy or hypometabolism using undirected GD [33, 34, 44]. The current study is therefore novel in demonstrating transsynaptic transmission directly on mouse pathology data, on a macroscopic basis along white matter fiber tracts (Tables 1 and 2; Figs. 1, 2, 3, 4, 5 and 6). This is the first study to our knowledge to mathematically demonstrate that whole brain macroscopic spatiotemporal tau pathology patterns are dependent upon anatomical connectivity and therefore fit the prion-like hypothesis of propagation [42]. We would also like to note that here all our analyses were performed with undirected networks, despite the availability of directional information in the mouse connectome. Our rationale behind this decision is that there is evidence from both patient studies [33] and assessments looking at the level of synapses [41] that transsynaptic tau spread is bidirectional. Given prior evidence for bidirectional transsynaptic and transregional tau transmission and a dearth of evidence for such transmission to be limited to only either afferent or efferent projections, we used bidirectional networks rather than impute directionality assumptions. However, assessing directional biases in tau transmission is important and should be the subject of future studies.

### Anatomic connectivity from the seed region is a stronger predictor of tau pathology progression than genetic proximity or regional gene expression profile

We specifically tested whether the spatiotemporal pattern of proteinopathy progression resulting from an exogenously inoculated and known seed region is more strongly predicted by anatomic connectivity or regional gene expression profile. The question of how much does gene expression contribute to pathology propagation vis a vis connectivity has been a debate in the tau pathology transmission field, as some mouse and clinical studies [11, 12] report results emphasizing the role of regional gene expression profile in determining regional susceptibility to tau pathology. Meanwhile other clinical research [33, 34, 44] points to the high predictive power the brain’s anatomic connectivity network has for recapitulating the spatiotemporal development of tau proteinopathy. Accumulating postmortem and mouse bench studies point to definite trans-synaptic propagation of tau. The highest accumulations of tau are often found at the synapse [41], at both pre and post synaptic terminals [16]. Tau pathology after exogenously seeding certain regions often exhibits enhanced deposition in axonally proximal regions, while sparing spatially proximal regions [2, 9, 10, 32], indicating that axonal projections, rather than spatial proximity, is the relevant mediator of spread.

To test this, we first performed a model-free statistical analysis involving only the reported seed region and assessed the association of tau severity in all brain regions with their proximity to the seed region. “Proximity” was defined in 3 ways: connectivity, gene expression similarity and spatial distance. We found that connectivity with reported seed regions, as given in the MBCA by axonal volume (Oh, et al., 2014), is the best biological correlate with regional pathology severity (Figs. 1 and 2). Both gene expression profile similarity with seed region (Table 1; Fig. 1) and higher absolute regional expression of tau aggregation and transcription promoting genes (Table 1; Fig. 2) failed to correlate as strongly with regional pathology as did connectivity. Spatial proximity was never a strong predictor of proteinopathy severity.

This kind of proximity-to-seed analysis is suggestive, but does not capture the full extent of wider, ongoing pathology progression. Therefore, we next implemented the mathematical Network Diffusion (ND) model, which was previously shown to accurately capture ongoing connectome-mediated spread in humans [33]. By applying this ND model to the mouse mesosclae anatomical connectome we test, in a regionally unbiased manner, the whole-brain macroscopic ramification of tau pathology transmission, and compare it against alternate predictors given by gene expression patterns. Thus, we built a ND model on the gene similarity network to model the hypothesis that tau transmission between two regions is facilitated by the similarity in their molecular signatures rather than anatomic connectivity. We also built a similar ND model involving spatial distance between brain regions, which would test for spatial spread. We found that, across all exogenously seeded studies, transregional spread on the anatomic connectome is a better predictor of spatiotemporal tau pathology development than spatial proximity or gene expression similarity, whether general or tau-specific genetic proximity networks (Table 2). ND using the brain’s connectivity network also outperforms the absolute levels of regional gene expression of tau-associated and noradrenergic neurotransmission-related genes, even when the predictive value of the seed region or a baseline pathology measurement are factored out (Table 2).

Our work therefore corroborates hypotheses of tau pathology development that lend primacy to the role of the brain’s anatomic connectivity network, and specifically implicate transsynaptic tau propagation as the most likely mechanism of spread from an exogenous seed region. Our data do not support a strong role for tau aggregation related genes [12] and the noradrenergic system [35] in determining regional vulnerability beyond the seeded region(s). Prior work characterizing regional expression of purported risk factors in prion and other degenerative disease agrees with our analysis, finding no consistent correlation between risk-factor expression level and regional degree of degeneration [22].

### The role of connectivity-based transmission in non-exogenously seeded models

Gene expression profile similarity with regions already exhibiting baseline tau pathology, across tau aggregation and transcription related genes as well as noradrenergic related genes, was a better correlate of regional pathology severity and the spatial pattern of tau pathology than was connectivity (Table 3). However, this result appeared potentially artefactual given the widespread pathology predictions in non-study-selected regions given only by ND using gene expression similarity, but not connectivity, networks (Fig. 5a). This concern was confirmed when all 426 ABA regions were included in our analyses, rather than only the study selected regions; in this reanalysis ND using the anatomic connectivity network was the only model to significantly improve upon baseline pathology (Table 3). Furthermore, ND using the connectivity network was a strong and significant predictor of regional tau pathology severity using all 426 ABA regions in a Multivariate Linear Model in the non-seeded dataset [17], while regional gene expression levels of neither tau or noradrenergic related genes was at any timepoint (Table 3, bottom section; Fig. 5b).

When we combined ND using the connectivity network and baseline pathology into a multivariate linear model using regional expression patterns of several specific genes as the other tau pathology predictors, a major AD risk factor gene [12] known to increase CSF tau, Bace1 [36], a gene known to promote tau hyperphosphorylation in cultured cells [39], Hs3st2, and the gene specifically necessary for norepinephrine synthesis, Dbh [35]. We found no evidence that absolute regional expression of any of these individual genes corresponded with regional susceptibility to tau pathology, but found that ND using the connectivity network was a strong and significant predictor of regional pathology vulnerability, despite controlling for the effect of baseline pathology (Fig. 5c). We further identified genes from our tau and noradrenergic related gene sets that were differentially expressed in regions exhibiting baseline pathology in the non-seeded dataset [17] but found that their regional expression levels did not reproduce the regional tau staging observed in the data (Fig. 5d). Our results suggest that even in non-exogenously seeded mouse tau pathology datasets, pathology spread is determined by more by connectivity than differences in regional gene expression.

Prior work using gene expression profile to explain regional vulnerability to tau pathology focused on the regions exhibiting earliest proteinopathy rather than subsequent propagation ([11, 12]; Hyman, et al., 1984; [28]), whether using the suite of tau aggregation promoting genes [12] or noradrenergic neurotransmission related genes [27]. Our results consistently show that connectivity is the key determinant of ongoing tau propagation and regional vulnerability once pathology has initiated. However, given the especially strong correlation between regional expression of our specific gene sets and regional pathology in our unseeded dataset, we believe our present results indicate a role for region-intrinsic factors in determining regions most likely to initiate tau pathology, in line with the major conclusions from [12]. Therefore, the current study does not rule out a role for regional gene expression profile (and other cell-dependent factors) in determining the location of tau pathology initiation, but demonstrates that once proteinopathy is apparent, regional vulnerability towards developing pathology is driven more by connectivity.

## Additional files

Additional file 1: Table S1. A list of genes used in the specific tau aggregation and expression factor related genes and noradrenergic neurotransmission related genes. The first column lists the gene abbreviations, the second lists the full gene name denoting basic function, and the third column gives the appropriate citation. **Table S2.** Regression and Multivariate Linear Models run with all 426, rather than only per-study selected regions. The entries under the “Bivariate Correlations” row correspond to the ΔR obtained from running the ND model with each row’s network from reported seedpoint. The four entries after the “Multivariate Linear Model” row represent the t-values and *p*-value thresholds obtained from ND model predictions or summed regional expression predictions after they were input as independent predictors into a Multivariate Linear Fit Model. *** *p* < 0.001, ** *p* < 0.01, * *p* < 0.05. (DOCX 132 kb)

Additional file 2: Figure S1. Per study r-value chart and scatterplots for connectivity, gene expression profile, and spatial proximity with reported seed regions. (a) Bar chart of r-values, per study, between regional tauopathy data and proximity with the reported seed region in connectivity, gene expression profile, and spatial distance networks. We also show scatterplots of the relationship between proximity with the reported seed region across each network, as indicated by the title above each scatterplot, and regional tau pathology data from (b) DSAD homogenate injected P301S mice and (c) CBD homogenate injected P301S mice from [4], (d) P301S mice injected in the hippocampus and (e) caudoputamen with synthetic tau fibrils from [19], as well as hTau Alz17 mice injected with P301S purified tau tangles in the hippocampus from [9]. **Figure S2.** Per study r-value chart and scatterplots of regionally summed gene expression across tau aggregation and transcription promoting genes, as well as noradrenergic neurotransmission related genes. (a) Bar chart of r-values for connectivity proximity with reported seed regions, empirical seed regions, and the regionally summed gene expression values with regional tau pathology data. We also show the scatterplots depicting the relationship between the regionally summed gene expression levels with data from (b) DSAD homogenate injected P301S mice and (c) CBD homogenate injected P301S mice from [4], (d) P301S mice injected in the hippocampus and (e) caudoputamen with synthetic tau fibrils from [19], as well as hTau Alz17 mice injected with P301S purified tau tangles in the hippocampus from [9]. **Figure S3.** Scatterplots and βt curves for each of the relationships between ND modeling using connectivity, gene expression profile similarity, and spatial distance networks with regional tau pathology data, run from reported seedpoints using only study selected regions. The panels are the βt curves for end state tau deposition and regional slope of tauopathy increase, in that order, as well as the scatterplots for end state tau deposition on the top and regional slope of tauopathy increase on the bottom. They are presented in the following order according to study: (b) DSAD homogenate injected P301S mice and (c) CBD homogenate injected P301S mice from [4], (d) P301S mice injected in the hippocampus and (e) caudoputamen with synthetic tau fibrils from [19], as well as hTau Alz17 mice injected with P301S purified tau tangles in the hippocampus from [9]. **Figure S4.** Scatterplots and βt curves for each of the relationships between ND modeling using connectivity, gene expression profile similarity, and spatial distance networks with regional tau pathology data, run from reported seedpoints using all 426 ABA regions. The panels are the βt curves for end state tau deposition and regional slope of tauopathy increase, in that order, as well as the scatterplots for end state tau deposition on the top and regional slope of tauopathy increase on the bottom. They are presented in the following order according to study: (b) DSAD homogenate injected P301S mice and (c) CBD homogenate injected P301S mice from [4], (d) P301S mice injected in the hippocampus and (e) caudoputamen with synthetic tau fibrils from [19], as well as hTau Alz17 mice injected with P301S purified tau tangles in the hippocampus from [9]. **Figure S5.** Scatterplots for the correlation between data from the non-seeded mouse dataset obtained from [17], with ND modeling across networks and timepoints as well as regionally summed gene expression with final measured timepoint of regional tauopathy severity; analysis here is done using both only study selected regions and all 426 ABA regions. (a) The beta-t parameter optimization curves at 4 months, 6 months, and 8 months using ND modeling with connectivity and gene expression networks, with analysis done using only study selected regions. (b) The attendant scatterplots related to the beta-t parameter curves above at the final (8 month) timepoint. (c) Scatterplot of regional expression of specific tau and noradrenergic related gene sets with regional tau pathology, using only study selected regions in the analysis. (d) The beta-t parameters optimization curves at 4 months, 6 months, and 8 months using ND modeling with connectivity and gene expression networks, with analysis done using all 426 ABA regions. (e) The attendant scatterplots using the curves for the final (8 month) timepoint. (f) Scatterplot of regional expression of specific tau and noradrenergic related gene sets with regional tau pathology, using all 426 ABA regions in the analysis. (PDF 1.14 mb)
