# Supplementary material for: Connectivity, not region-intrinsic properties, predicts regional vulnerability to progressive tau pathology in mouse models of disease
Source: Acta Neuropathol Commun. 2017 Aug 14;5:61. doi: 10.1186/s40478-017-0459-z (PMC5556602; doi:10.1186/s40478-017-0459-z)
Supplement: Supplementary file 1 — A list of genes used in the specific tau aggregation and expression factor related genes and noradrenergic neurotransmission related genes. The first column lists the gene abbreviations, the second lists the full gene name denoting basic function, and the third column gives the appropriate citation. Table S2. Regression and Multivariate Linear Models run with all 426, rather than only per-study selected regions. The entries under the “Bivariate Correlations” row correspond to the ΔR obtained from running the ND model with each row’s network from reported seedpoint. The four entries after the “Multivariate Linear Model” row represent the t-values and p-value thresholds obtained from ND model predictions or summed regional expression predictions after they were input as independent predictors into a Multivariate Linear Fit Model. *** p < 0.001, ** p < 0.01, * p < 0.05. (DOCX 132 kb) [file 40478_2017_459_MOESM1_ESM.docx]

| SPECIFIC GENES (TAU AGGREGATION AND TX FACTORS) | | |
| --- | --- | --- |
| Mapt | microtubule-associated protein tau | Freer, et al., 2016 |
| Bace1 | beta-site APP cleaving enzyme 1 | Freer, et al., 2016 |
| Mtf1 | metal regulatory transcription factor 1 | Bellingham, et al., 2008 |
| Hs3st2 | heparan sulfate (glucosamine) 3-O-sulfotransferase 2 | Freer, et al., 2016 |
| Hspa1l | heat shock protein 1-like | Freer, et al., 2016 |
| Hspb8 | heat shock protein 8 | Freer, et al., 2016 |
| Hspb1 | heat shock protein 1 | Freer, et al., 2016 |
| Cryab | crystallin, alpha B | Freer, et al., 2016 |
| Prnp | prion protein | All datasets used |
| Ap2s1 | adaptor related protein complex 2, sigma 1 | Freer, et al., 2016 |
| Ap2a2 | adaptor related protein complex 2, alpha 2 | Freer, et al., 2016 |
| Ap2b1 | adaptor related protein complex 2, beta 1 | Freer, et al., 2016 |
| Fos | fos AP-1 transcription factor subunit | Bellingham, et al., 2008 |
| Jun | jun AP-1 transcription factor subunit | Bellingham, et al., 2008 |
| NORADRENERGIC RELATED NEUROTRANSMISSION RELATED GENES | |  |
| Dbh | dopamine beta hydroxylase | Mather & Harley, 2016 |
| Maob | monoamine oxidase B | Mather & Harley, 2016 |
| Maoa | monoamine oxidase A | Mather & Harley, 2016 |
| Aldh1a1 | aldehyde dehydrogenase family 1, subfamily A1 | Mather & Harley, 2016 |
| Aldh2 | aldehyde dehydrogenase 2, mitochondrial | Mather & Harley, 2016 |
| Aldh1l1 | aldehyde dehydrogenase 1 family, member L1 | Mather & Harley, 2016 |
| Aldh1a2 | aldehyde dehydrogenase family 1, subfamily A2 | Mather & Harley, 2016 |
| Adra1d | adrenergic receptor, alpha 1d | Mather & Harley, 2016 |
| Adra2a | adrenergic receptor, alpha 2a | Mather & Harley, 2016 |
| Adra2b | adrenergic receptor, alpha 2b | Mather & Harley, 2016 |
| Adrbk2 | adrenergic receptor kinase, beta 2 | Mather & Harley, 2016 |
| Adra1a | adrenergic receptor, alpha 1a | Mather & Harley, 2016 |
| Adrb1 | adrenergic receptor, beta 1 | Mather & Harley, 2016 |
| Slc6a2 | solute carrier family 6 (neurotransmitter transporter, noradrenalin), member 2 | Mather & Harley, 2016 |

**Supplementary Table 1**. A list of genes used in the specific tau aggregation and expression factor related genes and noradrenergic neurotransmission related genes. The first column lists the gene abbreviations, the second lists the full gene name denoting basic function, and the third column gives the appropriate citation.

| **SUPPLEMENT TABLE 2** | **Measure** | **Boluda DSAD (6 Mo)** | **Boluda CBD (6 Mo)** | **Iba Hipp. Inj. (6 Mo)** | **Iba Str. Inj. (6 Mo)** | **Clavaguera (15 Mo)** |
| --- | --- | --- | --- | --- | --- | --- |
| **Mouse Model** | / | P301S | P301S | P301S | P301S | Alz17 |
| **Infusate** | / | DSAD Homogenate | CBD Homogenate | Synthetic Tau Fibrils | Synthetic Tau Fibrils | P301S Purified Tau |
| **Seed Region** | / | CA1 & V1 | CA1 | CA1 & CA3 | Caudoput. | Hippocampus |
| **ND USING ALL 426 ABA REGIONS (BIVARIATE CORRELATIONS)** | | | | | | |
| **Connectivity, Deposition** | ΔR | 0.26 | 0.16 | 0.27 | 0.21 | 0.02 |
| **Connectivity, Slope** | ΔR | 0.25 | 0.17 | 0.35 | 0.17 | 0.06 |
| **Spatial, Deposition** | ΔR | 0.02 | 0.00 | 0.00 | 0.00 | 0.00 |
| **Spatial, Slope** | ΔR | 0.01 | 0.00 | 0.00 | 0.00 | 0.00 |
| **General Gene, Dep.** | ΔR | 0.00 | 0.00 | 0.00 | 0.01 | 0.00 |
| **General Gene, Slope** | ΔR | 0.00 | 0.00 | 0.01 | 0.00 | 0.00 |
| **Specific Gene, Dep.** | ΔR | 0.00 | 0.00 | 0.00 | 0.00 | 0.00 |
| **Specific Gene, Slope** | ΔR | 0.00 | 0.00 | 0.00 | 0.00 | 0.00 |
| **Noradren. Gene, Dep.** | ΔR | 0.00 | 0.00 | 0.00 | 0.00 | 0.00 |
| **Noradren. Gene, Slope** | ΔR | 0.00 | 0.00 | 0.00 | 0.00 | 0.00 |
| **ND-CONNECTIVITY VS REGIONAL GENE EXPRESSION (MULTIVARIATE LINEAR MODEL)** | | | | | | |
| **Connectivity** | T-Stat | 8.30*** | 6.68*** | 17.26*** | 2.47* | 5.08*** |
| **Seed or Baseline** | T-Stat | -1.69 | -1.49 | -1.84 | 1.83 | 2.79** |
| **Summed Specific Gene Ex.** | T-Stat | 0.78 | -1.78 | -0.66 | 1.65 | 0.99 |
| **Summed Noradren. Gene Ex.** | T-Stat | -0.54 | 1.07 | 1.07 | 3.96*** | 1.72 |

**Table S2**. Regression and Multivariate Linear Models run with all 426, rather than only per-study selected regions. The entries under the “Bivariate Correlations” row correspond to the ΔR obtained from running the ND model with each row’s network from reported seedpoint. The four entries after the “Multivariate Linear Model” row represent the t-values and p-value thresholds obtained from ND model predictions or summed regional expression predictions after they were input as independent predictors into a Multivariate Linear Fit Model. *** p < 0.001, ** p < 0.01, * p < 0.05.
