# Supplementary figures and images for: Connectivity, not region-intrinsic properties, predicts regional vulnerability to progressive tau pathology in mouse models of disease
Source: Acta Neuropathol Commun. 2017 Aug 14;5:61. doi: 10.1186/s40478-017-0459-z (PMC5556602; doi:10.1186/s40478-017-0459-z)

# Per-Study Proximity Analysis

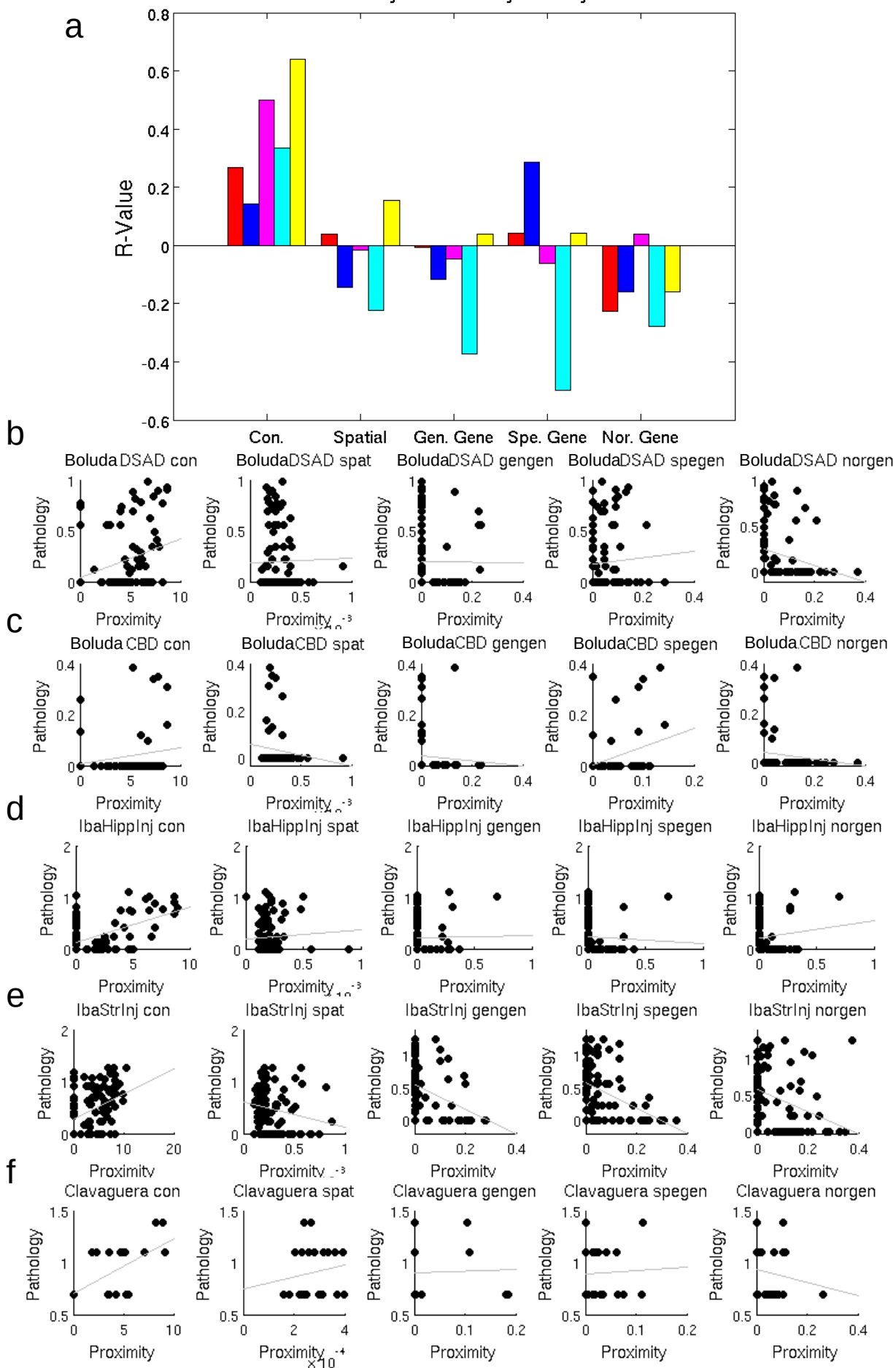

a

## Per Study Con. Proximity V Absolute Gene Ex.

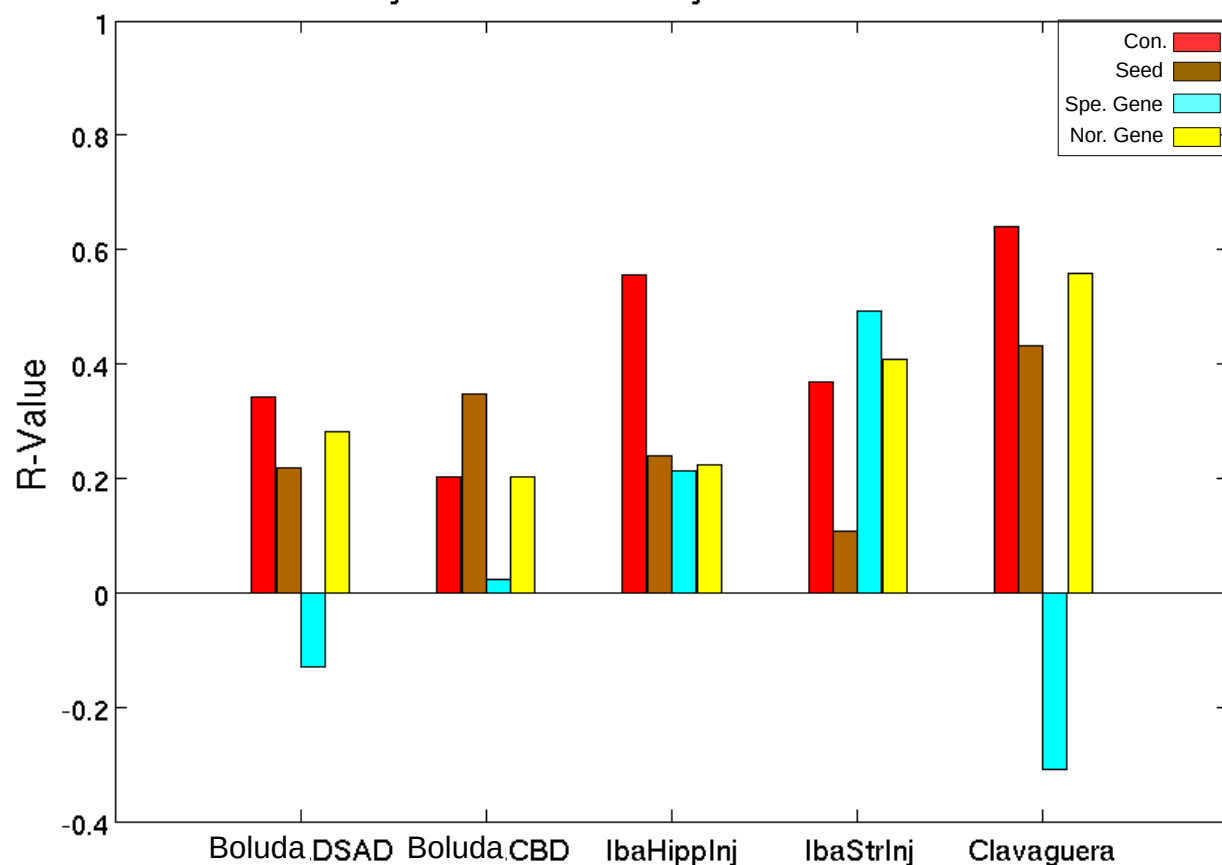

b

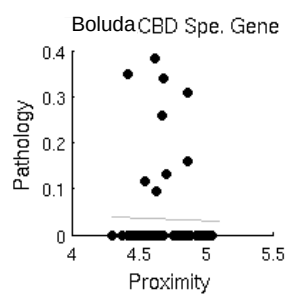

c

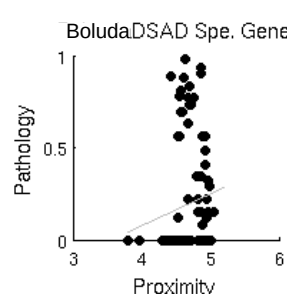

d

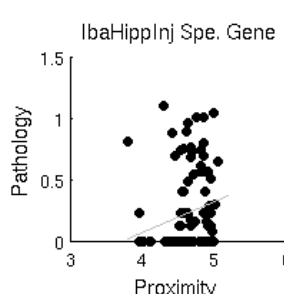

e

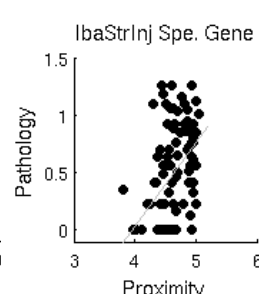

f

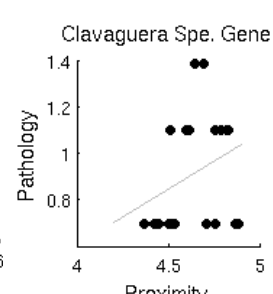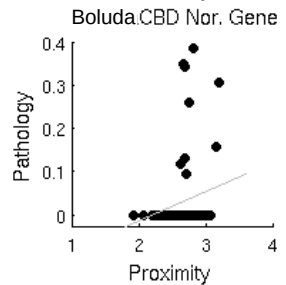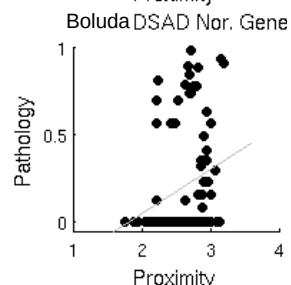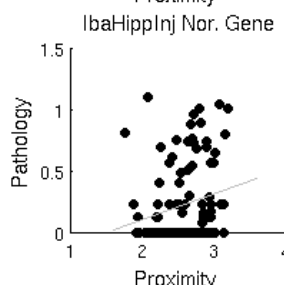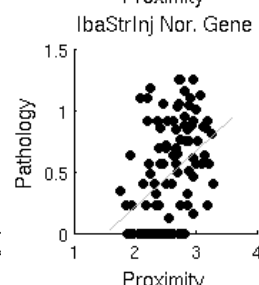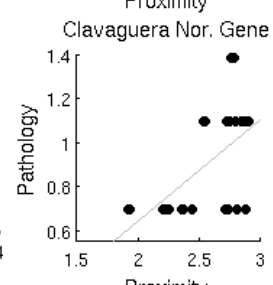

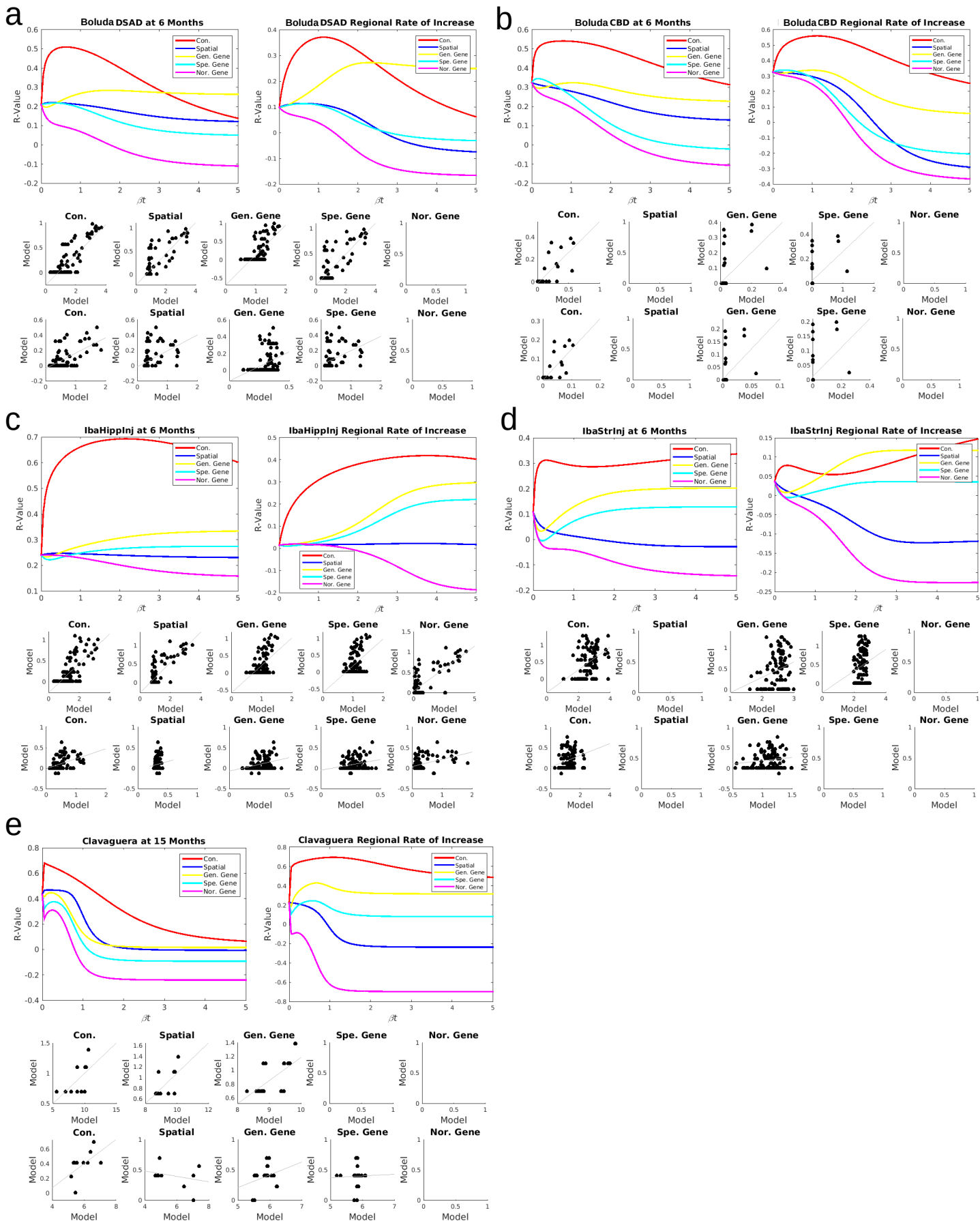

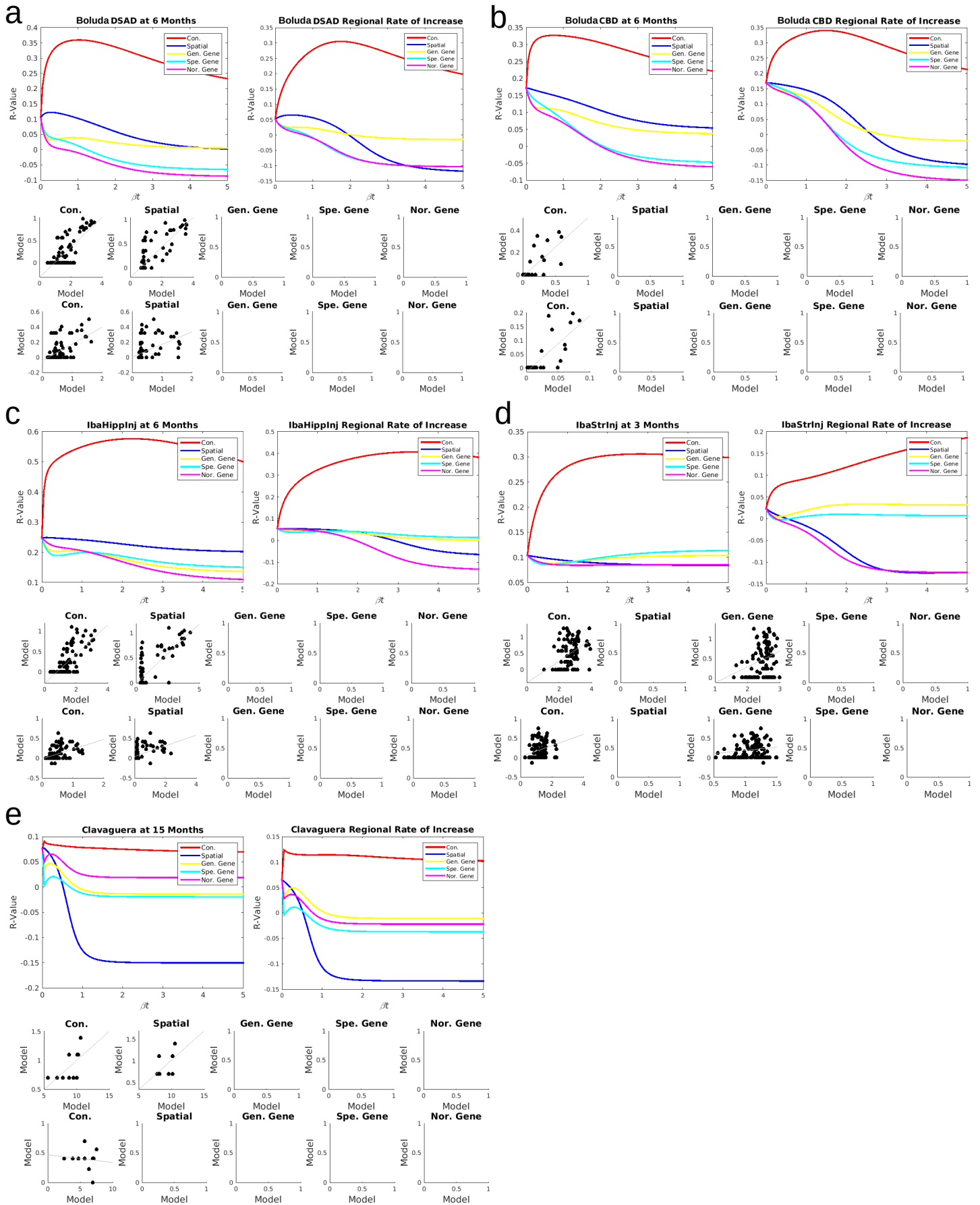

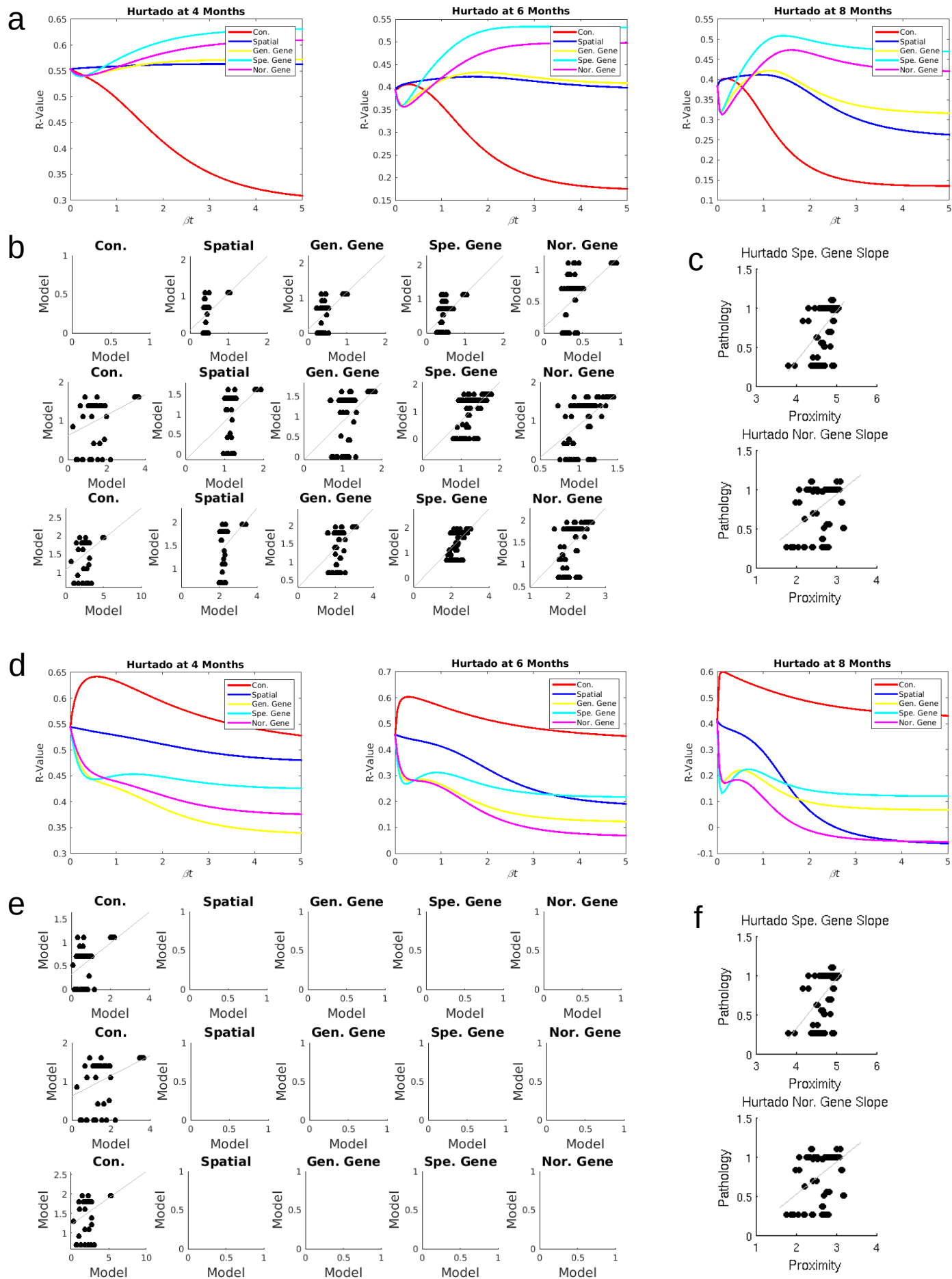

Supplement: Supplementary file 2 — Per study r-value chart and scatterplots for connectivity, gene expression profile, and spatial proximity with reported seed regions. (a) Bar chart of r-values, per study, between regional tauopathy data and proximity with the reported seed region in connectivity, gene expression profile, and spatial distance networks. We also show scatterplots of the relationship between proximity with the reported seed region across each network, as indicated by the title above each scatterplot, and regional tau pathology data from (b) DSAD homogenate injected P301S mice and (c) CBD homogenate injected P301S mice from [4], (d) P301S mice injected in the hippocampus and (e) caudoputamen with synthetic tau fibrils from [19], as well as hTau Alz17 mice injected with P301S purified tau tangles in the hippocampus from [9]. Figure S2. Per study r-value chart and scatterplots of regionally summed gene expression across tau aggregation and transcription promoting genes, as well as noradrenergic neurotransmission related genes. (a) Bar chart of r-values for connectivity proximity with reported seed regions, empirical seed regions, and the regionally summed gene expression values with regional tau pathology data. We also show the scatterplots depicting the relationship between the regionally summed gene expression levels with data from (b) DSAD homogenate injected P301S mice and (c) CBD homogenate injected P301S mice from [4], (d) P301S mice injected in the hippocampus and (e) caudoputamen with synthetic tau fibrils from [19], as well as hTau Alz17 mice injected with P301S purified tau tangles in the hippocampus from [9]. Figure S3. Scatterplots and βt curves for each of the relationships between ND modeling using connectivity, gene expression profile similarity, and spatial distance networks with regional tau pathology data, run from reported seedpoints using only study selected regions. The panels are the βt curves for end state tau deposition and regional slope of tauopathy increase, in [file 40478_2017_459_MOESM2_ESM.pdf]
